# Too Wordy, Too Long and Too Cluttered: User Acceptance Testing, Co-Design, and Early Beta Testing to Adapt the Daily Health Coach for Young Australian Women

**DOI:** 10.3390/nu18193150

**Published:** 2026-09-24

**Authors:** Rajshri Roy, Isabella Hamilton, Demera Khan, Mariah Issa, Jasmin Alami, Jessica A. Malloy

**Affiliations:** 1 Nutrition and Dietetics, Susan Wakil School of Nursing and Midwifery, Faculty of Medicine and Health, The University of Sydney, Sydney, NSW 2050, Australia; iham8831@uni.sydney.edu.au (I.H.); dkha0438@uni.sydney.edu.au (D.K.); mariah.issa@sydney.edu.au (M.I.); jessica.malloy@sydney.edu.au (J.A.M.); 2 Charles Perkins Centre, The University of Sydney, Sydney, NSW 2006, Australia; 3 School of Public Health, Faculty of Medicine and Health, The University of Sydney, Sydney, NSW 2050, Australia; 4 TUM School of Life Sciences, Technical University of Munich, 85354 Freising, Germany; jasmin.alami@tum.de

**Keywords:** young women, social media, digital health, nutrition, co-design, user acceptance testing, usability testing, body image, health promotion, cultural adaptation

## Abstract

**Background/Objectives:** The Daily Health Coach (DHC) is a 12-week Instagram-based nutrition and wellbeing programme originally co-designed and tested in New Zealand to deliver evidence-based, weight-neutral content on nutrition, movement, body image, and wellbeing. This formative, mixed-methods adaptation study examined the acceptability of DHC New Zealand content for young Australian women and identified refinements to improve relevance, engagement, and safety before effectiveness evaluation. **Methods:** Using the CODES framework, we conducted three adaptation phases: user acceptance testing, co-design workshops, and early beta testing. Following data-quality screening, 291 young women aged 18–24 years who completed all 12 survey sections were included in Phase 1. Participants rated 84 Instagram-style posts for understanding and usefulness and provided open-ended feedback. Twelve young women participated in co-design workshops, and seven adapted artefacts were beta-tested with eight target users using cognitive walkthrough and think-aloud methods. Quantitative findings were examined descriptively, with sensitivity analyses undertaken to assess robustness to response-quality and sample-composition concerns. **Results:** Participant-level mean understanding was 3.99 ± 0.75/5, usefulness was 3.89 ± 0.74/5, and the combined score was 7.87 ± 1.47/10. Post-level combined means ranged from 7.66 to 8.11/10; all 84 posts met a ≥7.5 descriptive benchmark adapted from prior message-testing work, although this is an interpretive aid rather than a validated cut-off. Sensitivity analyses showed a similar overall pattern: ratings were higher in a higher-confidence response-quality sample (*n* = 199; 8.34 ± 1.20/10), while four posts fell marginally below 7.5 when analysis was restricted to participants not reporting Aboriginal and/or Torres Strait Islander identity (*n* = 101). Qualitative feedback identified six refinement areas: content features, engagement strategies, clarity, educational accuracy, relevance, and miscellaneous concerns. Co-design and beta testing generated four adaptation priorities: tone, design, engagement, and understanding. **Conclusions:** DHC New Zealand content was generally well understood and favourably rated but required targeted refinement for the Australian context. Findings provide formative guidance for final adaptation and subsequent effectiveness evaluation.

## 1. Introduction

Young adulthood is a transitional period in which education, work, living arrangements, relationships, finances, and food environments can change rapidly, shaping health behaviours and wellbeing trajectories across the life course [1]. For young women, these transitions occur within a digital environment where social media platforms are now routine sources of nutrition, fitness, body image, and wellbeing information. This creates both risk and opportunity: social media can rapidly disseminate misinformation, unrealistic ideals, and commercialised wellness messages, but it can also be used to deliver accessible, evidence-based health promotion in formats that young people already use [2,3,4].

Young women are a priority audience for digital health promotion because appearance-focused and influencer-led content has been associated with body image concerns, self-objectification, and disordered eating risk [2,5]. In an international web-based survey, women aged 16–25 years reported high levels of body image distress [4], and Australian youth survey data suggest that body image concerns remain prominent among young women [6]. Prior Daily Health Coach (DHC) work in New Zealand also found that time spent on social media was positively associated with body image disturbance among young women [3]. These findings underscore the need for health promotion content that is not only accurate, but also framed in ways that avoid reinforcing shame, comparison, or diet-culture messaging.

Digital platforms can support health promotion because they allow repeated exposure to concise, visual, and interactive messages at scale [7,8,9,10]. Social media interventions have shown promise for influencing physical activity, diet-related behaviours, and mental wellbeing, particularly when content is designed around audience needs and platform conventions [8,9]. However, translating evidence into social media content is not a simple formatting task. For young audiences, engagement depends on whether a message feels relevant, visually clear, credible, actionable, and emotionally safe. These considerations are especially important for nutrition content, where messages can easily become prescriptive, moralising, or inadvertently triggering.

The DHC is a co-designed Instagram-based health promotion programme for young women. It delivers daily posts, reels, stories, and direct messages through a weight- and diet-neutral lens, with content addressing nutrition, physical activity, mental wellbeing, social wellbeing, body image, and digital health literacy. The New Zealand DHC programme was developed with young women and demonstrated feasibility, high acceptability, strong satisfaction, and preliminary improvements in body image disturbance and digital health literacy [11,12]. Building on this evidence, the present study adapted the DHC for young Australian women.

Cultural adaptation is necessary when digital health interventions are translated across settings because language, health systems, food availability, social norms, platform use, and cultural references can alter how content is interpreted and used [13]. Participatory and co-design approaches are widely recommended to ensure that interventions are relevant, acceptable, and contextually safe for target users [14,15]. This paper reports the first three phases of a CODES-guided adaptation process: user acceptance testing (UAT) of DHC New Zealand posts, co-design workshops to interpret feedback and refine content priorities, and early beta testing/adaptation to examine how message framing, tone, and design influence understanding and engagement with nutrition content.

The present study is therefore positioned as a methodological adaptation study rather than an effectiveness evaluation. The DHC was originally co-designed and tested with young women in New Zealand, where prior studies established its feasibility, acceptability and preliminary effects on digital health literacy, body image and wellbeing outcomes [11,12]. However, translating a social media-based nutrition intervention into a new country is not a simple process of reusing existing posts. Digital nutrition content must be assessed for local relevance, cultural and food-system fit, language, tone, credibility, emotional safety and practical usefulness, particularly when delivered to young women who may already be exposed to appearance-focused health misinformation online [3,16]. This paper therefore reports formative evidence from the first three phases of Australian adaptation: user acceptance testing of the New Zealand DHC content, co-design workshops to interpret and prioritise refinements, and early beta testing to examine how message framing, tone, visual design and engagement features shaped understanding and perceived usefulness.

### Aims

This study aimed to describe the first three phases of DHC adaptation for young women in Australia and to identify practical guidance for more effective digital nutrition and health messaging. The research questions were:

How acceptable are the current DHC New Zealand posts to young Australian women?

What adaptations are required to ensure that DHC content is relevant, engaging, understandable, and culturally appropriate for young Australian women?

How do message framing, tone, and design influence early perceptions of adapted DHC content, and what practical guidelines can inform final content development?

## 2. Materials and Methods

### 2.1. Design

This study used an iterative, mixed-methods, multi-phase design following the CODES framework (Co-designing for Optimal Digital Engagement and Scalability). CODES is a stepwise adaptation approach that combines quantitative user acceptance testing, qualitative sense-making, co-design, beta testing, and later real-world evaluation (currently under review). The current paper reports Phases 1–3: (1) UAT via online survey; (2) online co-design workshops with target users; and (3) early beta testing and adaptation using cognitive walkthrough and think-aloud methods (Figure 1).

### 2.2. Ethics

Ethics approval was granted by the University of Sydney Human Research Ethics Committee on 5 August 2025 for a three-year period (Reference number: 2025/HE00139). Participants provided electronic informed consent before taking part in the online survey, workshops, or interviews; Phase 1 consent was obtained online within REDCap. Participants self-reporting a current active diagnosis of anorexia nervosa or bulimia were excluded as an eligibility/safety screen rather than a validated diagnostic assessment to reduce potential risk from exposure to nutrition- and body-image-related content. Because the study materials addressed food, eating and body image, the following safeguards were in place: support-service contact details (for example, the Butterfly Foundation National Helpline and Lifeline) were provided in the participant information statement and on the survey landing page; participants could skip any item and could exit the survey, workshop or interview at any point without giving a reason and without penalty; researcher contact details were provided for follow-up; workshop and interview facilitators were briefed to pause or end a session and to re-provide support-service details if a participant appeared distressed.

### 2.3. Phase 1: User Acceptance Testing of DHC New Zealand Posts

The purpose of Phase 1 was to assess the acceptability, clarity and usefulness of DHC New Zealand content for young women in Australia. Cultural relevance was not assessed with a quantitative item; it was explored through open-ended survey feedback and subsequently through the Phase 2 co-design discussions. The source content comprised DHC posts from the 12-week New Zealand programme. Survey data were collected and managed using REDCap (https://caribdata.org/redcap/, accessed 22 September 2025), a secure, web-based software platform designed to support data capture and management for research studies [17]. For survey administration, one post from each day of each intervention week was selected, resulting in 84 Instagram-style static posts covering seven recurring weekly themes: Myth Busting, Simple Swaps, Things You Should Know, The Other Important Stuff, Real Talk, Healthy Navigation, and Recipe Sharing.

Young women aged 18–24 years residing in Australia and with sufficient English proficiency to complete an online survey were recruited via targeted Instagram advertisements and digital flyers. The survey was hosted in REDCap and was available from 6 to 8 August 2025. Participants were excluded if they identified as male, were outside the target age range, or reported a current active diagnosis of anorexia nervosa or bulimia. Female, non-binary, and genderqueer participants were eligible.

The UAT survey was organised into 12 sections corresponding to the 12-week programme. For each post, participants completed four items: two five-point Likert-scale questions assessing understanding and usefulness, one tick-all-that-apply item assessing age appropriateness, and one open-ended question inviting feedback or suggestions for improvement. Responses to the age-appropriateness item were collected but are not analysed or reported in this paper; age appropriateness is therefore not treated as a Phase 1 outcome. Demographic data included age, gender, education level, Aboriginal and Torres Strait Islander status, and postcode. Residential postcode was mapped to the Australian Bureau of Statistics Socio-Economic Indexes for Areas (SEIFA) 2021 Index of Relative Socio-economic Advantage and Disadvantage (IRSAD), using national deciles for Postal Areas [18]. Completion time was estimated at approximately 30 min after preliminary REDCap testing. The survey was informed by usability-testing principles [19,20]; it was not the validated System Usability Scale, and no validated SUS items were administered.

### 2.4. Phase 2: Co-Design Workshops with Target Users

Phase 2 used UAT findings as a sense-making input for co-design workshops with young Australian women. Workshop materials, moderation guides, and activities were adapted from the original DHC co-design scaffold and revised using early survey findings. Participants were recruited at the end of the UAT survey and through targeted Instagram recruitment. Interested participants were provided with participant information and consent forms and completed a short Zoom screening interview to confirm eligibility, identify access barriers, and reduce the likelihood of automated or bot participation.

Four online workshops were conducted over four weeks, with each workshop offered twice at times convenient for participants. Workshops were delivered via Zoom to support participation across Australia. Each session lasted up to 90 min and was capped at fewer than eight participants to support equitable contribution. Facilitators used structured prompts and group activities but limited their intervention during discussion to allow participants to guide interpretation and prioritisation of content refinements, referred to as study-specific touchpoints. Workshop objectives are summarised in Table 1.

### 2.5. Phase 3: Early Beta Testing and Adaptation Process

Early beta testing used cognitive walkthrough and think-aloud methods, established usability approaches for examining how users interpret, navigate and respond to digital health content or interfaces [21,22]. Phase 3 translated Phase 2 touchpoints into a small sample of adapted DHC artefacts and examined initial user perceptions of those artefacts. Seven adapted posts were developed for testing: three static posts and four reels. Adaptations focused on message framing, tone, caption structure, visual layout, cultural relevance, and the degree to which nutrition content was actionable without becoming prescriptive or appearance focused.

Participants who had engaged in co-design workshops were invited to beta testing, and additional participants were recruited through unpaid posts on the study Instagram page and a study website pop-up. One-on-one interviews were conducted via Zoom using a study-specific protocol informed by cognitive walkthrough and think-aloud methods. Participants completed a series of content review tasks and were asked to verbalise their thoughts as they viewed adapted posts. Interviews lasted up to 90 min, with an option for follow-up if needed. This phase was formative and was designed to refine content rather than evaluate intervention effectiveness.

### 2.6. Data Cleaning and Analysis

A total of 613 REDCap records were received. Of these, 188 did not complete all 12 survey sections, leaving 425 complete records for response-quality screening. A further 134 complete records were excluded during the original study-team data-cleaning process based on response-quality criteria, including implausibly fast survey completion and email verification failure, resulting in a final primary analytic sample of 291 participants. All 291 participants completed the 12 survey sections and had complete understanding and usefulness ratings for all 84 posts. Record identifiers and email addresses were also checked for duplication, with no duplicate identifiers or normalised email addresses remaining in the analytic dataset.

Understanding and usefulness were each assessed using five-point Likert response options ranging from Strongly agree to Strongly disagree. Because REDCap coded Strongly agree as 1 and Strongly disagree as 5, responses were reverse-scored before analysis so that higher values represented more favourable ratings (5 = Strongly agree; 1 = Strongly disagree). Understanding and usefulness scores were summarised separately, and the two scores were summed to produce a combined score ranging from 2 to 10 for each post. Quantitative analysis was primarily descriptive, reflecting the formative purpose of the study. For each participant, mean understanding, usefulness and combined scores were calculated across the 84 posts and summarised using means and standard deviations, medians and interquartile ranges. Post-level means were also calculated across participants. A combined score of ≥7.5/10 was used as a descriptive benchmark only. The threshold was adapted from prior digital message-testing work with young people [23]; it has not been validated against any external criterion and is not a clinical or psychometric cut-off. It is reported to aid interpretation of the distribution of scores rather than to classify individual posts as acceptable or unacceptable.

Education and other demographic characteristics were summarised descriptively. Inferential subgroup comparisons were not undertaken because the study was not designed or powered to test demographic differences and several education groups contained very small and unequal numbers of participants.

Three analytic samples were defined. The primary cleaned sample (*n* = 291) comprised all records that completed the 12 survey sections and passed the response-quality screening described above. A higher-confidence response-quality sample was then derived by applying only criteria that had not already been used in the primary cleaning, so that no exclusion criterion was applied twice. Records were excluded from this higher-confidence sample if they showed complete straight-lining across all 168 understanding and usefulness rating items (*n* = 8 removed), an exact duplicate 168-item rating pattern shared with another participant (*n* = 6 additional records removed), or highly unusual temporal duplication, defined as sharing identical REDCap completion timestamps with the same other respondent for at least six of the 12 survey sections (*n* = 78 additional records removed). Where a record met more than one criterion, it was counted once only, under the first criterion met, so that exclusions were mutually exclusive and summed to the total removed. This excluded 92 records and produced a higher-confidence response-quality sample of *n* = 199. A third sample applied these same response-quality criteria and was additionally restricted to participants who selected “No” to Aboriginal and/or Torres Strait Islander identity, giving *n* = 75; the one participant with missing Indigenous-status data was excluded from this restricted analysis. Primary descriptive outcomes were recalculated in each sample, and the overlap between samples is reported in Section 3.2.

Response-quality indicators were audited across the primary cleaned sample. We examined: (i) the distribution of elapsed survey completion time, summarised as median, interquartile range and full range; (ii) straight-lining, defined as selecting an identical response option across all 168 understanding and usefulness ratings; (iii) within-person response variability, quantified as the standard deviation of each participant’s 168 Likert ratings; (iv) postcode validity, checked against the current list of Australian residential postcodes; and (v) duplicate and timestamp-pattern checks, comprising duplicate record identifiers, duplicate normalised email addresses, and shared completion timestamps across respondents. The REDCap project did not retain IP address or geolocation data. To test whether lower-quality responding biased ratings in a particular direction, mean combined scores were compared descriptively between records flagged by these indicators and records not flagged.

The unexpectedly high proportion of participants reporting Aboriginal and/or Torres Strait Islander identity was treated as a potential sampling or data-quality anomaly and was not interpreted as representative of young Australian women. A separate sensitivity analysis therefore recalculated the primary acceptability outcomes among participants who did not report Aboriginal and/or Torres Strait Islander identity (*n* = 101) to determine whether the principal findings were dependent on this subgroup.

Open-ended UAT responses were analysed using qualitative content analysis. Open-ended feedback was provided by 39 respondents, contributing 422 coded segments across six themes. Comments were coded by I.H. and D.K., comprising 2 coders. Coding was initially inductive and subsequently reviewed deductively against the research questions, with an initial coding frame developed from the first 50 comments and then applied to the full set of comments. Twenty per cent of comments were independently double-coded, and disagreements were resolved by discussion to consensus. Reported frequencies refer to coded segments derived from open-ended responses rather than participants, and a single respondent could contribute to more than one theme; the six themes comprise 422 coded segments in total.

For Phases 2 and 3, transcripts were cleaned against audio recordings, de-identified, and analysed using an adapted thematic approach informed by Braun and Clarke and focus group analysis guidance [24,25,26]. Codes were first organised inductively, then reviewed deductively in relation to the research questions. Themes were consolidated into adaptation touchpoints and practical recommendations for final DHC Australia content development.

With respect to reflexivity, the research team comprised nutrition and dietetics academics and postgraduate research students, all of whom were women and 5 were within or close to the target age range, and 2 of the researchers had prior involvement in the New Zealand DHC programme. The team’s orientation towards weight-neutral nutrition and digital health promotion shaped the framing of workshop prompts and the interpretation of participant feedback, and these assumptions were examined through team debriefing. Sample size for Phases 2 and 3 was not determined by data saturation, which would not be an appropriate criterion for an iterative co-design process. Sufficiency was instead judged against the adaptation purpose: recruitment continued until sessions were generating converging, artefact-level detail specific enough to specify concrete content changes, within the practical constraints of the adaptation timeline.

## 3. Results

### 3.1. Participants and Study Flow

Of 613 REDCap records received, 322 were removed in total: 188 records did not complete all 12 survey sections, and a further 134 complete records were excluded at response-quality screening. The final primary analytic sample comprised 291 participants, all of whom completed ratings for the 84 DHC posts. Demographic completeness ranged from 92% to 100%. Participants were predominantly female (*n* = 288, 99.0%), with one non-binary participant, one genderqueer participant and one missing response; mean age was 21.38 years (SD 1.81). Aboriginal and/or Torres Strait Islander identity was reported by 189/290 participants (65.2%), with one response missing. Participant flow across phases is shown in Figure 2 and demographic characteristics are summarised in Table 2.

For Phase 2, 27 participants initially expressed interest in co-design. Twenty-one completed a short screening interview; six did not proceed to screening. We did not document the specific reasons for non-screening for these six individuals. Of the 21 screened, nine were excluded because they did not meet the inclusion criteria, did not respond after screening, or were assessed as likely automated responses, leaving 12 young women who participated in the co-design workshops (Workshop 1: *n* = 12; Workshop 2: *n* = 12; Workshop 3: *n* = 10; Workshop 4: *n* = 8). For Phase 3, eight participants completed one-on-one beta-testing interviews; six were recruited from the co-design workshops and two via social media. Four young women contributed to all three phases.

### 3.2. Phase 1: Quantitative User Acceptance Testing

Participant-level mean understanding was 3.99 ± 0.75/5 and mean usefulness was 3.89 ± 0.74/5. The mean combined understanding and usefulness score was 7.87 ± 1.47/10, with a median of 8.32 (IQR 6.18–9.04) and a participant-level range of 4.88–10.00. Overall, 196/291 participants (67.4%) had an individual mean combined score ≥7.5. Across the 84 individual posts, mean combined scores ranged from 7.66 to 8.11/10, and all 84 post-level means were ≥7.5. Understanding was consistently rated slightly more favourably than usefulness, suggesting that although participants generally understood the content, there remained greater scope to improve its perceived relevance and actionability.

At the level of individual ratings rather than means of means, 18,480/24,444 understanding ratings (75.6%) and 17,430/24,444 usefulness ratings (71.3%) were Agree or Strongly agree. Post-level standard deviations for the combined score ranged from 1.70 to 2.04. The highest-scoring post was Week 1, Post 7, Recipe Sharing (“Cauliflower and Chickpea Curry”) (combined mean 8.11 ± 1.70/10), and the lowest-scoring was Week 10, Post 4, The Other Important Stuff (“No Right Way to Eat”) (combined mean 7.66 ± 1.95/10). Mean ± SD combined scores for each of the seven recurring content themes are presented in Table 3.

Response-quality indicators in the primary cleaned sample were as follows. Median elapsed completion time was 40.6 min (IQR 27.3–55.8; range 12.4–1085.4). Straight-lining across all 168 understanding and usefulness ratings was observed in 8 participants (2.7%). The within-person standard deviation across the 168 ratings had a median of 0.66 (IQR 0.50–0.91). Rather than introducing an arbitrary definition of “minimal variability”, we report the full distribution and complete straight lining separately. A total of 290/291 participants (99.7%) provided a four-digit postcode entry; one postcode entry was invalid in format. Postcodes were matched to 2021 ABS Postal Area SEIFA data to derive socioeconomic deciles. Eighty-three records (28.5%) shared identical REDCap completion timestamps with the same other respondent for at least six of the 12 survey sections. IP address and geolocation fields were not collected. For the response-quality sensitivity analysis, 92/291 records were flagged by at least one of the prespecified additional indicators: complete straight-lining, an exact duplicate 168-item response pattern, or highly unusual temporal duplication. These flagged records had a mean combined score of 6.86 ± 1.51/10, compared with 8.34 ± 1.20/10 among the 199 unflagged records, indicating that the response patterns identified as lower confidence were associated with lower, rather than higher, acceptability ratings. Aboriginal and/or Torres Strait Islander identity was reported by 189/290 respondents (65.2%; one response missing), a proportion substantially higher than expected from Australian population data. Accordingly, these data were not interpreted as representative, and a sensitivity analysis was conducted among participants who did not report Aboriginal and/or Torres Strait Islander identity (*n* = 101). In this subset (*n* = 101), the mean combined score was 7.79 ± 1.58/10 and post-level means ranged from 7.42 to 8.30. For comparison, participants who did report Aboriginal and/or Torres Strait Islander identity (*n* = 189) had a mean combined score of 7.91 ± 1.42/10 (understanding 4.01 ± 0.73/5; usefulness 3.91 ± 0.71/5), a descriptive difference of 0.12 points higher than the non-Indigenous-identifying subgroup.

Eighty of 84 posts (95.2%) remained ≥7.5, while all 84 remained ≥7.0. The four posts below 7.5 were narrowly below the benchmark (7.42–7.49), indicating that the overall pattern of favourable ratings was not dependent on the anomalous subgroup. These subgroup figures are reported only to establish whether responses from this group behaved differently within the dataset. Given the implausibly high proportion reporting this identity, the absence of any culturally specific measure, and the online recruitment method, they carry no interpretation regarding Aboriginal and Torres Strait Islander young women.

Outcomes are reported for three samples: (i) the primary cleaned sample, *n* = 291; (ii) the higher-confidence response-quality sample, *n* = 199; and (iii) the higher-confidence sample additionally restricted to participants not reporting Aboriginal and/or Torres Strait Islander identity, *n* = 75. Sample (ii) comprised 199 of the 291 primary-sample participants (68.4%); sample (iii) was wholly nested within sample (ii) and comprised 37.7% of it. In sample (ii), mean understanding was 4.23 ± 0.60/5, usefulness 4.11 ± 0.62/5, and the combined score 8.34 ± 1.20/10, with post-level combined means ranging from 8.10 to 8.59 and 84/84 posts at or above 7.5. In sample (iii), the combined score was 7.91 ± 1.45/10, with post-level means ranging from 7.48 to 8.41 and 83/84 posts at or above 7.5.

### 3.3. Phase 1: Qualitative Feedback from User Acceptance Testing

Open-ended feedback provided explanatory context for the quantitative acceptability ratings. Six themes were identified, with participants describing specific features that influenced the clarity, usefulness, relevance and perceived safety of DHC content. Representative quotations and the corresponding interpretation and adaptation response are presented in Table 4. Content features were the most common category (*n* = 129, 30.5%), followed by engagement strategies (*n* = 116, 27.5%), miscellaneous concerns (*n* = 77, 18.3%), content clarity (*n* = 44, 10.4%), educational accuracy (*n* = 34, 8.1%), and relevance (*n* = 22, 5.2%). The quote used in the title-“too wordy, too long, and too cluttered”-captures the dominant usability challenge: the DHC content was acceptable, but needed to be more concise, visual, and tailored to the Australian audience.

### 3.4. Phase 2: Co-Design Findings

Co-design workshops enabled participants to interpret UAT findings, expand on why posts were useful or not useful, and prioritise adaptations. Analysis identified four overarching themes: tone, design, engagement, and understanding. Design was the most frequently coded theme (*n* = 365 coded segments, 51.0%), followed by engagement (*n* = 225, 31.4%), understanding (*n* = 86, 12.0%), and tone (*n* = 40, 5.6%). Although tone was the least frequently coded theme, it was conceptually important because participants repeatedly emphasised that the emotional quality of the message shaped whether they trusted, ignored, or resisted the content.

Participants described effective tone as personable, supportive, and non-judgemental. They wanted content that sounded like it came from a credible but relatable account, rather than a school lesson, influencer advertisement, or clinical warning. One participant noted, “If you actually want to educate or engage with younger people, you need to use personality.” Participants also emphasised sensitivity around body image, weight, and food choice. Messages were viewed more positively when they promoted agency and wellbeing without implying that health is only for thin bodies or that food choices are moral failures.

Design findings extended the UAT feedback about clutter. Participants wanted a clear visual hierarchy, consistent graphics, fewer competing colours, captions that did not simply repeat the post, and format decisions that matched the content’s purpose. They viewed reels as particularly useful where demonstration, voice, or movement could simplify information. As one participant explained, “I just think it’s easier to listen to people than read a long post.” Engagement findings focused on actionability and interaction: participants were more likely to stay with content that offered concrete examples, low-cost swaps, local food contexts, resource links, and prompts that invited reflection rather than compliance. Understanding depended on plain language, explanation of unfamiliar terms, and the ability to tell quickly why the post mattered.

### 3.5. Phase 3: Early Beta Testing and Adaptation Guidelines

Phase 3 translated UAT and co-design findings into adapted static posts and reels. The beta-testing process demonstrated that message framing, tone, and design functioned together rather than independently. A concise caption could still feel unhelpful if the visual hierarchy was confusing; a visually appealing post could still be rejected if the tone felt judgemental; and evidence-based nutrition information could still be misunderstood if the key action was not made explicit. The most successfully adapted artefacts therefore combined a simple primary message, a supportive frame, a clear visual pathway, and a practical next step.

Table 5 These guidelines are intended to support the final co-creation and refinement phase before formal trial evaluation. They prioritise clarity, safety, relevance, and platform-native engagement while preserving the evidence-based and weight-neutral intent of the original DHC programme. Phase 3 findings are reported through the design principles and participant feedback arising from testing. Final before-and-after artefacts will be reported once the co-creation cycle has been completed.

## 4. Discussion

### 4.1. Principal Findings

This study combined user acceptance testing, co-design workshops, and early beta testing to generate formative evidence for adapting the DHC from New Zealand for young Australian women. In the primary UAT analysis, participants generally rated the content favourably for understanding and usefulness, and all 84 post-level mean scores exceeded the ≥7.5 descriptive benchmark. Because that benchmark is an interpretive aid adapted from prior message-testing work rather than a validated threshold, the more informative observation is the narrow spread of post-level means alongside substantial variation between participants. However, participant-level scores showed meaningful variability, and sensitivity analyses undertaken in response to the unexpectedly high proportion of participants reporting Aboriginal and/or Torres Strait Islander identity provided a more cautious picture. When analyses were restricted to participants who did not report Aboriginal and/or Torres Strait Islander identity, 80 of 84 posts remained above the ≥7.5 benchmark, with the remaining four only marginally below it. Findings were also maintained in the more stringent response-quality sample, in which ratings were higher (8.34 ± 1.20 vs. 7.87 ± 1.47/10). Because the excluded records rated the content less favourably (6.86 ± 1.51/10), the primary estimate is, if anything, conservative rather than inflated by low-quality responding. Together, these analyses suggest that the overall pattern of favourable ratings was robust, while reinforcing that the findings should be interpreted as formative evidence of acceptability rather than definitive evidence that every item was equally acceptable across all young Australian women. The consistently lower usefulness-than-understanding ratings indicate that content can be clear at the level of comprehension while still requiring refinement to become personally relevant, engaging, and actionable. For social media health promotion, acceptability is therefore necessary but insufficient; messages must also be framed and designed in ways that fit the target audience’s contexts, platform habits, and emotional safety needs.

Qualitative feedback focused predominantly on how the health messages were delivered rather than on rejection of the underlying health information. They wanted posts that were shorter, visually cleaner, more practical, more local, more personable, and more sensitive to the risks of body-image and diet-culture messaging. This aligns with prior DHC research showing the value of co-design with young women and the importance of digital health literacy, body image safety, and experience-informed content development [3,11,12]. It also aligns with broader evidence that digital health engagement depends on more than exposure: users must perceive content as relevant, trustworthy, usable, and worth interacting with [8,9,10,27].

### 4.2. Implications for Social Media-Based Nutrition Messaging

The results highlight three implications for nutrition communication on social media. First, clarity should be treated as a design outcome, not simply a writing outcome. Participants responded negatively to clutter, small or low-contrast text, repeated caption content, and too much information in a single post. This suggests that evidence-based nutrition content requires visual hierarchy and format discipline if it is to be processed quickly in a scroll-based environment.

Second, usefulness is shaped by contextual relevance. Young women aged 18–24 years are not a homogeneous audience: they differ in study and work commitments, living arrangements, budgets, relationships and food autonomy. Life-stage diversity may plausibly contribute to differences in perceived usefulness, although this was not directly tested in the present study. Future DHC content should therefore use flexible scenarios and examples rather than assuming a single lifestyle or food environment. Adaptation from New Zealand to Australia likewise required more than changing isolated words or visual features. Participants identified New Zealand-specific terminology and asked for locally recognisable language, relatable food examples and trusted Australian sources, with practical relevance further shaped by cost-of-living pressures and varying food autonomy. Future Australian content should therefore reflect local food availability and terminology, culturally diverse food practices, Australian referral contexts, and visible links to credible local evidence—requests for sources suggesting that credibility is itself part of contextual adaptation.

Third, tone is central to safety. Participants wanted the account to have personality, but not to sound performative, patronising, authoritarian, or like it was trying too hard to be relatable. They also wanted nutrition content to avoid triggering pathways associated with calorie counting, thinness, food rules, and body comparison. For young women, evidence-based nutrition messaging must be paired with careful framing so that health promotion does not inadvertently reproduce the harms it seeks to counter.

### 4.3. Implications for Adaptation and Co-Design Methodology

This study demonstrates the value of sequencing methods. The UAT survey provided breadth, allowing many participants to assess all 12 weeks of posts. Co-design workshops then provided depth and sense-making, helping explain why some posts felt useful, unclear, or culturally misaligned. Beta testing translated those insights into artefact-level decisions and generated practical content rules. This staged process is consistent with recommendations for participatory design and cultural adaptation of digital health interventions [13,14,15]. It also shows why adaptation should be documented transparently: without a clear record of how feedback becomes design change, co-design risks becoming consultative rather than transformative.

The smaller samples used in the co-design and beta-testing phases should be interpreted according to their purpose. These phases were designed to provide depth, explanation, and iterative feedback rather than population-level representativeness. Accordingly, the perspectives of the 12 co-design participants and eight beta-testing participants may not reflect the full diversity of the broader UAT sample, particularly across socioeconomic, cultural, and life-stage circumstances. Their contribution was therefore to help interpret UAT findings and identify plausible design priorities, rather than to establish universally applicable content preferences. The practical guidelines generated through these phases should be regarded as provisional design principles that require further testing when the complete Australian DHC content library is implemented and evaluated with a larger and more diverse sample.

It is also important to distinguish the CODES framework from a behaviour-change framework. CODES structured the participatory adaptation process and guided how target users contributed to content refinement; it was not used to specify behavioural determinants or mechanisms of action. The DHC content evaluated in the present study was evidence-informed and developed through prior co-design [28]. Still, this phase did not prospectively map individual messages to a single formal behaviour-change theory [29]. Accordingly, the present findings relate to acceptability, usability, relevance, and adaptation rather than mechanisms of behaviour change. Before effectiveness evaluation, future work should explicitly map the final DHC Australia content to an appropriate behaviour-change framework and identify the hypothesised mechanisms linking specific content features to outcomes such as digital health literacy, dietary behaviour, and body-image-related wellbeing. Self-determination theory is one example of a framework that could plausibly suit an autonomy-supportive, weight-neutral programme of this kind [29], but the framework should be selected because of the final content and specified outcomes rather than assumed in advance.

### 4.4. Strengths and Limitations

A key strength of this study was its staged mixed-methods design, integrating quantitative ratings, open-ended feedback, co-design discussions, and early beta testing. The final UAT sample included 291 participants who completed all 12 survey sections, providing broad formative feedback across the complete 84-post content library. The subsequent co-design and beta-testing phases added explanatory depth by enabling participants to interpret survey findings and respond directly to adapted content. The use of response-quality and demographic sensitivity analyses further strengthened interpretation of the UAT findings by testing whether the principal conclusions were dependent on aspects of the sample that raised data-quality concerns.

Several limitations should be acknowledged. The UAT was an online convenience sample and should not be considered representative of all young Australian women. Most notably, the proportion of respondents self-identifying as Aboriginal and/or Torres Strait Islander was substantially higher than expected from population estimates. Although this characteristic was not itself treated as evidence of an invalid response, the discrepancy raised concerns regarding sample composition and possible residual low-quality online responses. We therefore undertook sensitivity analyses restricted both by response-quality indicators and by Aboriginal and Torres Strait Islander status. Relatedly, no inference should be drawn from these data about the relevance or acceptability of the DHC for Aboriginal and Torres Strait Islander young women. Establishing whether and how this content is appropriate for that population would require dedicated, culturally appropriate, Indigenous-led engagement and co-design, which was outside the scope of this online survey.

The online recruitment approach also limited independent verification of participant identity and eligibility and created challenges in detecting automated or AI-assisted survey responses. Data-quality procedures included removal of incomplete records, duplicate or test entries, bounced or invalid email addresses, implausibly fast completions, and other indicators of low-quality responding, followed by additional sensitivity analyses. Nevertheless, it is not possible to exclude all residual misclassification in an anonymous online environment. The Phase 2 and Phase 3 samples were also small and purposively used to obtain depth rather than representativeness, and their perspectives may not capture the diversity of the wider UAT population. Finally, this study assessed perceived acceptability, understanding, usefulness, and early usability rather than actual social media engagement, dietary behaviour, or health outcomes; these outcomes require evaluation in the subsequent trial phase.

### 4.5. Future Research

The next phase of DHC Australia adaptation should apply the practical guidelines generated in this study to co-create a complete, updated content library with young Australian women. The final content library should also be explicitly mapped to a behaviour-change framework, with hypothesised mechanisms of action specified before effectiveness testing. The practical tone, design, engagement, and understanding principles identified in the smaller co-design and beta-testing samples should then be evaluated prospectively to determine whether they remain acceptable and useful in a larger and more socioeconomically and culturally diverse cohort. Subsequent work should evaluate whether adapted content improves engagement, digital health literacy, body image outcomes, and diet-related behaviours in a formal trial. Future research should also examine whether tailored or adaptive content pathways improve perceived usefulness across different life circumstances, including study/work status, living arrangement, cultural background, food autonomy, and socioeconomic context. Finally, there is a need for clearer standardised reporting of UAT and co-design methods for digital health interventions to improve comparability across studies.

## 5. Conclusions

The DHC New Zealand content was generally well understood and favourably rated by young Australian women, while formative testing identified important opportunities to improve usefulness, engagement, cultural relevance, and safety. Quantitative findings were broadly maintained across sensitivity analyses. However, variation between participants and the anomalous demographic composition of the UAT sample warrant cautious interpretation. Qualitative feedback showed that young women preferred content that was concise, visually clear, practical, locally relevant, evidence-supported, and delivered in a supportive, non-judgemental tone. Co-design and early beta testing translated these findings into provisional principles for adapting digital nutrition and health messaging from New Zealand to Australia. These principles now require application to the complete DHC Australia content library and prospective evaluation in a larger and more diverse sample before conclusions can be drawn about effectiveness or broader transferability.

## Figures and Tables

**Figure 1:**
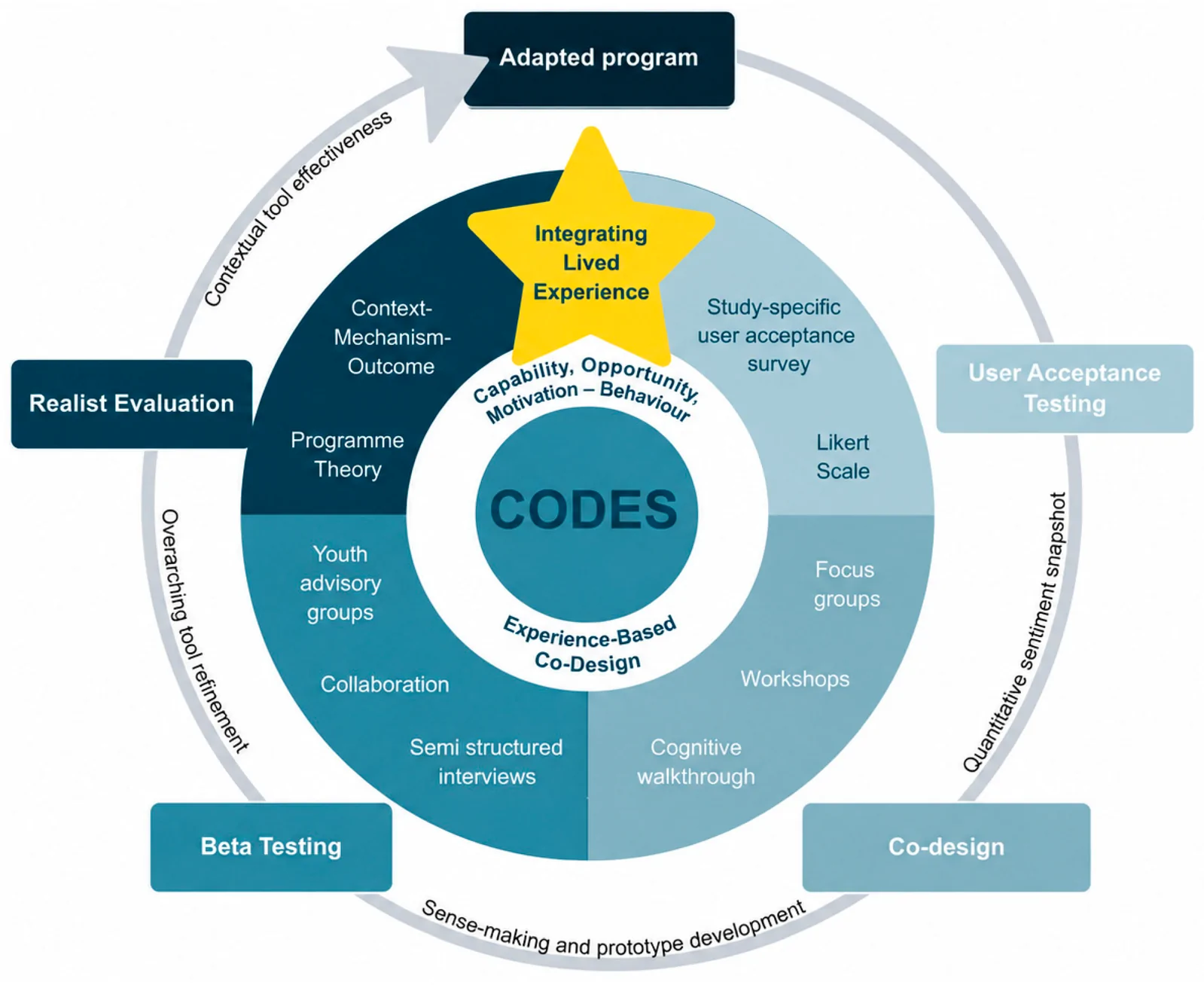
CODES framework for cultural adaptation of the Daily Health Coach. This paper reports the first three phases: user acceptance testing, co-design to refine output, and beta testing/adaptation of artefact samples.

**Figure 2:**
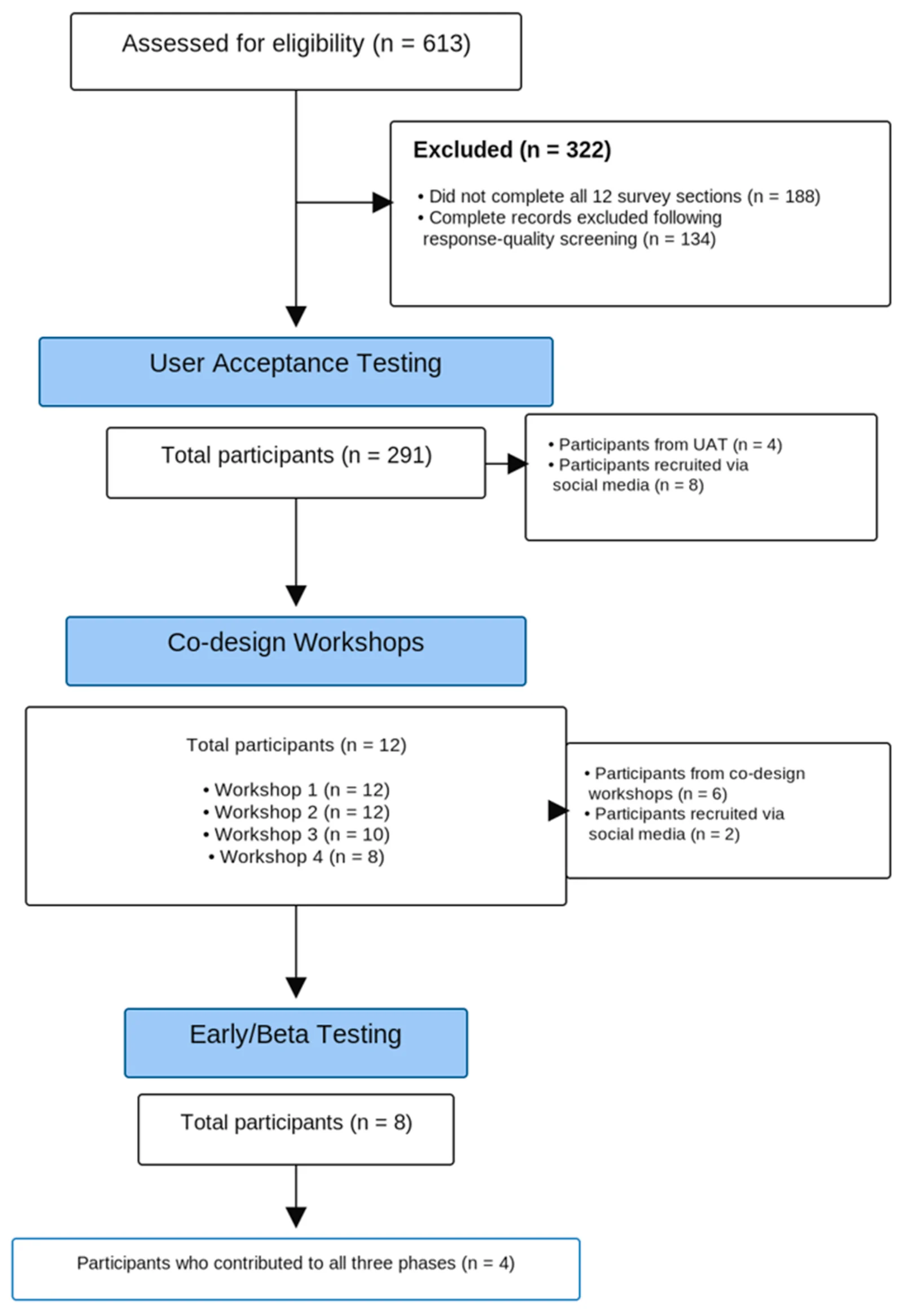
Participant flow across user acceptance testing, co-design workshops, and early beta/usability testing.

**Table 1:** Co-design workshop objectives, activities, and adaptation outputs.

Workshop	Objective	Core Activities	Actioned Adaptation
1. Health and wellbeing meanings	Understand how young women define health, wellbeing, healthy/unhealthy behaviours, confidence, barriers, and priorities.	Mentimeter prompts and facilitated discussion about health, wellbeing, barriers, and content needs.	Clarified the values and concerns that should underpin tone and framing.
2. Social media behaviours	Understand platform use, preferred content formats, exposure to health content, and engagement behaviours.	Discussion of most-used apps, time spent online, limits, preferred formats, and types of health content participants interact with.	Identified platform conventions and format preferences for DHC Australia.
3. Content reactions	Explore how health and wellbeing content, features, and design make young women feel.	Participants reviewed TikTok and Instagram examples, including reels and static posts, and discussed whether they would stay or swipe.	Identified design, visual, and emotional response cues that shape engagement.
4. DHC content sense-making	Understand how participants related to DHC New Zealand themes, posts, captions, and UAT findings.	Participants reviewed DHC New Zealand content, discussed likes/dislikes, Australian relevance, and agreement with UAT results.	Generated priorities for localisation, content simplification, and adapted artefact development.

**Table 2:** Phase 1 participant demographic summary.

Variable	Category	*n* (%) or Mean (SD)
Gender	Female	288 (99.0%)
	Non-binary	1 (0.3%)
	Genderqueer	1 (0.3%)
	Missing	1 (0.3%)
Age, years	Mean (SD)	21.38 (1.81)
Aboriginal and/or Torres Strait Islander identity ^a^	Yes	189/290 (65.2%)
	No	101/290 (34.8%)
	Missing	1 (0.3%)
Education level	Year 10	4 (1.4%)
	Year 12	25 (8.6%)
	Technical/TAFE	3 (1.0%)
	Undergraduate	197 (67.7%)
	Postgraduate	61 (21.0%)
	Other	1 (0.3%)
Socioeconomic status (SEIFA decile)	1–3	5%
	4–7	15%
	8–10	80%

^a^ Aboriginal and Torres Strait Islander identity was self-reported. Percentages for Aboriginal and/or Torres Strait Islander identity were calculated using participants with non-missing responses (*n* = 290); one participant had missing data. The unexpectedly high proportion observed in this convenience sample was treated as a sampling/data-quality anomaly and was not interpreted as representative of the Australian population; sensitivity analyses are reported separately.

**Table 3:** Mean ± SD combined scores for each of the seven recurring content themes.

Content Theme	Combined Score, Mean ± SD/10
Myth Busting	7.91 ± 1.52
Simple Swaps	7.86 ± 1.52
Things You Should Know	7.88 ± 1.48
The Other Important Stuff	7.88 ± 1.51
Real Talk	7.86 ± 1.48
Healthy Navigation	7.85 ± 1.51
Recipe Sharing	7.88 ± 1.51

Education groups were highly unequal in size: Year 10 (*n* = 4), Year 12 (*n* = 25), technical education (*n* = 3), undergraduate education (*n* = 197), postgraduate education (*n* = 61), and other education (*n* = 1). Mean combined scores were 7.26 ± 0.88, 8.12 ± 1.01, 8.71 ± 0.13, 7.94 ± 1.45, 7.56 ± 1.72, and 7.79, respectively. Given the very small and unbalanced subgroup sizes, these differences were treated descriptively, and no inferential conclusions were drawn.

**Table 4:** Thematic summary of open-ended user acceptance testing feedback.

Theme	Participant Quote	Interpretation	Resulting Adaptation
Content features	“Some photos/illustration of steps would be helpful” (ID 137); “you could add a short reel to make it easier” (ID 604).	Participants wanted information to be communicated through more visual, multimedia and practical formats rather than relying heavily on text.	Increase use of reels, recipe visuals, animation and step-by-step imagery where these formats can simplify or demonstrate information.
Engagement strategies	“Try using shorter, punchier titles to grab attention” (ID 78); “use emojis to make the post more visually engaging” (ID 93); “provide a link to resources/trusted accounts” (ID 331).	Participants valued concise hooks, platform-appropriate visual cues, clear calls to action and opportunities to access further information.	Use shorter openings and titles, purposeful platform-native engagement features and clear prompts or links to further resources.
Content clarity	“Too much information on the posts and difficult to read with all the different colours used” (ID 20); “slightly more concise, it covers good information but is a long read” (ID 331).	Dense text and competing visual elements reduced readability and made otherwise useful information harder to process quickly.	Reduce on-image text, simplify captions, strengthen visual hierarchy and use spacing and design to emphasise the primary message.
Educational accuracy	“So what is the research behind the ‘fasting’ fad” (ID 600); “link to studies and evidence” (ID 452).	Participants wanted clearer justification for nutrition claims, particularly for contested or unfamiliar topics, and visible signals of information credibility.	Provide plain-language explanations of the rationale behind key messages and incorporate links or cues directing users to credible evidence and trusted sources.
Relevance	“Does not take into account different people have different needs with food” (ID 137); “I think this would be more useful for people aged below 15, as it is a bit more childish” (ID 158). One participant also suggested replacing solely New Zealand-oriented language with an inclusive Australian reference: “kia ora and g’day!” (ID 587).	Some content was perceived as overly generic, mismatched to the intended life stage or insufficiently adapted from its original New Zealand context. Participants also cautioned against assuming that one nutrition message would suit all users.	Localise language, food examples and life-stage scenarios for young Australian women, while avoiding one-size-fits-all messaging and acknowledging variation in individual circumstances and needs.
Miscellaneous concerns	“People may be hesitant to follow a page which may expose their eating habits or difficulties” (ID 158); “may introduce the idea of calorie counting to someone who isn’t already doing this behaviour–potential negative effects?” (ID 331). Participants also requested “allergy substitutions or nut free suggestions” (ID 331).	Participants identified potential unintended harms relating to privacy, stigma, triggering dietary practices and inclusivity for people with food allergies or different dietary needs.	Strengthen content safety review, avoid unnecessarily introducing potentially harmful diet practices, incorporate inclusive food alternatives where appropriate, and consider privacy and stigma when designing engagement strategies.

**Table 5:** Practical adaptation guidelines for effective DHC Australia digital nutrition and health messaging.

Guideline	Rationale from Phases 1–3	Practical Application for DHC Australia	Examples
1. Lead with one clear takeaway	Posts were understandable but not always useful; dense posts were described as too long, wordy, or cluttered.	Use one headline message per slide/post; place supporting detail in captions, carousels, or optional links.	“There’s text on the side, in the middle, below, what do I focus on” “I’m used to fast paced content; our attention spans are conditioned for that”
2. Make nutrition advice actionable and contextual	Usefulness depended on whether participants could see how the advice applies to work, study, living at home, budgeting, or time pressure.	Use realistic Australian examples, low-cost options, pantry/freezer substitutions, and flexible prompts rather than idealised routines.	“Not in such a way that it is so professional and looks like you can’t cook it at home” “Different people have different needs” “I like this post because it feels realistic”
3. Use a supportive, non-judgemental, weight-neutral tone	Participants were sensitive to messages that could imply shame, calorie fixation, body comparison, or thinness as the goal.	Avoid moralising food language; frame content around energy, enjoyment, confidence, access, wellbeing, and self-compassion.	“made me feel a bit inadequate” “it almost made me feel guilty for not being like that”
4. Design for fast scanning before deeper learning	Design was the dominant co-design theme; users decide quickly whether to stay or swipe.	Use consistent templates, high-contrast text, fewer colours, clear hierarchy, white space, and platform-native visual cues.	“Aesthetics do matter, they have to be clear and clean” “The colours are really nice, they draw my eyes in” “I like how the background is plain”
5. Match format to content purpose	Participants wanted more reels and visuals, especially where processes or recipes were explained.	Use reels for demonstrations, recipes, and complex explanations; use static posts for simple reminders, myth-busting, and resource signposting.	“More information on the actual post rather than the caption” “its (preference for reel or static post) generally based on the content”
6. Signal credibility without overwhelming users	Participants asked for evidence, resources, and explanations, but also disliked overly long captions.	Include plain language “why it matters” statements, trusted links, and references in captions or bio, while keeping the main post concise.	“It feels more credible if it comes from a specific source” “Showing credentials would help” “It tries to make science approachable but maybe goes a bit too far”

## Data Availability

The datasets generated and analysed during the current study are not publicly available because they contain participant responses from formative qualitative and survey research, but de-identified data may be made available from the corresponding author upon reasonable request and subject to ethics approval.
